# Palladium nanoparticle-decorated reduced graphene oxide sheets synthesized using *Ficus carica* fruit extract: A catalyst for Suzuki cross-coupling reactions

**DOI:** 10.1371/journal.pone.0193281

**Published:** 2018-02-21

**Authors:** Jaculin Raiza Anasdass, Pandian Kannaiyan, Raghunathan Raghavachary, Subash C. B. Gopinath, Yeng Chen

**Affiliations:** 1 Department of Inorganic Chemistry, University of Madras, Chennai, India; 2 Department of Organic Chemistry, University of Madras, Chennai, India; 3 School of Bioprocess Engineering, Arau, Universiti Malaysia Perlis, Perlis, Malaysia; 4 Institute of Nano Electronic Engineering, Kangar, Universiti Malaysia Perlis, Perlis, Malaysia; 5 Department of Oral & Craniofacial Sciences, Faculty of Dentistry, University of Malaya, Kuala Lumpur, Malaysia; 6 Oral Cancer Research & Coordinating Center (OCRCC), Faculty of Dentistry, University of Malaya, Kuala Lumpur, Malaysia; Institute of Materials Science, GERMANY

## Abstract

We present a biogenic method for the synthesis of palladium nanoparticle (PdNP)-modified by reducing graphene oxide sheets (rGO) in a one-pot strategy using *Ficus carica* fruit juice as the reducing agent. The synthesized material was well characterized by morphological and structural analyses, including, Ultraviolet-Visible spectroscopy (UV-Vis), X-ray diffraction (XRD), Fourier transform infrared spectroscopy (FT-IR) and Transmission Electron Microscopy (TEM) and Raman spectroscopy. The results revealed that the PdNP modified GO are spherical in shape and estimated to be a dimension of ~0.16 nm. The PdNP/graphene exhibits a great catalytic activity in Suzuki cross-coupling reactions for the synthesis of biaryl compounds with various substrates under both aqueous and aerobic conditions. The catalyst can be recovered easily and is suitable for repeated use because it retains its original catalytic activity. The PdNP/rGO catalyst synthesized by an eco-friendly protocol was used for the Suzuki coupling reactions. The method offers a mild and effective substitute to the existing methods and may significantly contribute to green chemistry.

## Introduction

Palladium nanoparticles (PdNP) play a vital role in different fields, including sensors, catalysis, and as energy storage materials [1–5]. As a catalyst, palladium (Pd) has been commonly used in Heck, Suzuki, Stille and Sonogashira reactions for the formation of carbon-carbon bonds [6]. The benefit of PdNP as a heterogeneous catalyst for the production of pharmaceuticals, polymers, herbicides, and fine chemicals has advantages over the use of homogenous Pd catalysts due to their selectivity and stability. Additionally, they are cheaper, can be recycled and are very reactive [7–13].Because of good catalytic activity, PdNP is a subject of great interest and have the potential to be used as heterogeneous catalysts with unique properties.

To overcome several issues related to homogeneous Pd catalysts, many attempts have been made to stabilize and attach PdNP to heterogeneous supports, such as carbon [14–16], carbon nanotubes [17], alumina [18], silica [19–20], zeolite [21,22], clay [23], and zinc ferrite [24]. The carbon-mediated supports are attractive due to their stability in different media, low weight, abundance, and low reactivity toward support metals. These materials also minimize surface poisoning, simplify the recovery of the metal by burning, and serve as versatile structural allotropes [25–28].

Graphene is more commonly used as a stabilizer for PdNP than other carbon-based materials, for example, porous carbon and carbon nanotubes, because of their high availability, high corrosion resistance, low cost, and good dispersion in nature [29,30]. Graphene exhibits a single layer of conjugated sp^2^ carbon atoms and has received a substantial amount of attention recently because of exceptional mechanical and electronic properties and large surface area [31–35]. Graphene and its derivatives are widely used as adsorbents for removing textile dyes and separating oils and as a catalyst for the degradation of color dyes and organic pollutants. Various strategies have been proposed for the synthesis of PdNP-modified graphene oxide. Hydrazine [36], ethylene glycol [37], sodium borohydride [38], and sodium dodecyl sulfate [39] have been used as reducing agents to synthesis PdNP-functionalized graphene.

The development of methods for green syntheses of nanoparticles has become emerging areas of research. The main advantage of these methods is eliminating the use of toxic reducing agents and avoiding harsh experimental conditions. A few reports have demonstrated the synthesis of PdNP using green chemistry approaches [40–42]. In addition, few publications have shown the promising applications of PdNP synthesized by these methods. Some examples are, *Nasrollahzadeh et al*. reported a green synthesis of PdNP using *Hippophae rhamnoides Linn* leaf extract and their catalytic activity for the Suzuki–Miyaura coupling in water [43]. He also studied green synthesis of bimetallic (Pd/Fe_3_O_4_) nanoparticles using *Euphorbia condylocarpa M*. *bieb* root extract and their catalytic applications as magnetically recoverable and stable recyclable catalysts for the phosphine-free Sonogashira and Suzuki coupling reactions [44]. Khan *et al*. described a biogenic synthesis of palladium nanoparticles using *Pulicaria glutinosa* extract and their catalytic activity towards the Suzuki coupling reaction [45]. *Raju et al*. studied a green synthesis of palladium nanoparticles by *Sapindus mukorossi* seed extract and use in efficient room temperature Suzuki–Miyaura cross-coupling reaction [46]. *Sadaf et al*. reported a green synthesis of palladium nanoparticles mediated by black tea leaves (*Camellia sinensis*) extract: Catalytic activity in the reduction of 4-nitrophenol and Suzuki-Miyaura coupling reaction under ligand-free conditions [47]. PdNP modified rGO not only used for Suzuki coupling reaction alone. It has application in various studies such as sensors, nitophenol reduction, electrocatalytic and photocatalytic activity. It is also used for various organic reactions such as Heck, Sonogashira, Ullmann and Stille coupling reactions [48–50].

The *Ficus carica* fruit extract which we have taken up for our study contains high levels of polyphenols, flavonoids, and anthocyanins and exhibited the good antioxidant capacity. It is used as traditional medicine to treat various diseases such as gastrointestinal (colic, indigestion, loss of appetite, and diarrhea), respiratory (sore throats, coughs, and bronchial problems), and cardiovascular disorders and as anti-inflammatory and anti-spasmodic remedy. The fruits are also used as a mild laxative, expectorant, and diuretic. It is used as an aid in liver and spleen diseases. Due to these properties, the *Ficus carica* fruit was used for the synthesis of PdNP [51,52].

Poly(N-vinyl-2-pyrrolidone) (PVP) is among the most commonly employed capping ligands in the size and shape-controlled synthesis of metal nanoparticles. PVP-capped metal nanoparticles have been much explored as catalysts for liquid phase and gas-phase catalytic reactions, and their catalytic performance has been reported to be positively or negatively affected by chemisorbed PVP molecules. Generally, chemisorbed PVP ligands were found to poison the catalytic activity of metal nanoparticles by lowering the accessibility of free metal surfaces. This PVP molecule chemisorbs with its oxygen atom in the ring; for large PdNP capped with a small number of PVP molecules, each PVP molecule chemisorbs with its oxygen atom and a nitrogen atom in the ring. However, charge transfer always occurs from the chemisorbed PVP ligand to PdNP [53,54]. Since, the chemisorbed PVP ligands are used as a stabilizing agent for the synthesis of PdNP/rGO and showed the enhanced catalytic activity for Suzuki coupling reaction.

In the current study, we have chosen *Ficus carica* fruit extract as the reducing agent for synthesizing PdNP (Scheme I in S1 File). It is a rich source of beneficial bioactive flavonoid-based antioxidants such as quercetin and luteolin [55]. Since, the fruit extract was utilized as the reducing agent for the efficient synthesis of PdNP in a single step and simultaneously acts as an agent to reduce graphene oxide and the Pd salt (Scheme II in S1 File). We designed a novel, green strategy for the preparation of PdNP-embedded reduced graphene oxide (PdNP/rGO) using *Ficus carica* fruit extract as the reducing agent and PVP as the stabilizer to study its catalytic activity in aerobic Suzuki couplings. The catalyst was separated easily from the product and can be used repeatedly.

## Experimental

### Materials

Freshly grown *Ficus carica* fruits were obtained from the University of Madras Campus, Chennai. Palladium chloride (H_2_PdCl_4_), aryl halides, aryl boronic acids, sodium borohydride (NaBH_4_) and graphite powder were procured from Sigma-Aldrich, India. Potassium carbonate (K_2_CO_3_), potassium permanganate (KMNO_4_), hydrogen peroxide (H_2_O_2_), ethanol (EtOH), ethyl acetate (EtOAc), hexane, concentrated hydrochloric acid (con. HCl) and concentrated sulfuric acid (con. H_2_SO_4_) were obtained from SRL Chemicals, India. All glassware was rinsed with aquaregia (3:1 ratio of HCl:HNO_3_) and then washed thoroughly with double-distilled water.

#### Preparation of *Ficus carica* fruit extract and synthesis of PdNP

Approximately 10 g of freshly obtained *Ficus carica* fruit was washed and crushed using a mortar and pestle to obtain a wet paste. The paste was suspended in100 mL of double deionized water (ddw), and the filtered extract was stored at 5 °C. Approximately 2 mL of *Ficus carica* fruit extract was suspended in 1 mL of 1mM H_2_PdCl_4_, diluted to 5 mL with ddw, and incubated for 3 h at room temperature. The PdNP formed was stable at 5 °C. The mechanism for reduction of Pd ions using *Ficus carica* fruit extract is shown in scheme III in S1 File.

### Preparation of PdNP/rGO

Graphene oxide was produced by oxidizing graphite powder as outlined by the method of Hummers and Offeman [56]. Initially, the prepared rGO (200 mg) was mixed with distilled water (40 mL) and then sonicated (30 min) to prepare rGO sheets. The obtained mixture was placed in a round-bottomed glass container, and then 100 mg (0.563 mmol) of H_2_PdCl_4_ and 0.03 g of PVP added to the mixture. The container was fitted with a cooling condenser, and the temperature was raised to 80 °C. Then, 10 mL of *Ficus carica* fruit extract was poured and the mixture was placed for 24 h (80 °C) with a constant stirring. The obtained PdNPs/rGO was isolated as a black powder by filtration and thoroughly rinsed with ddw to eliminate additional *Ficus carica* fruit extract residue. The PdNPs/rGO was then suspended in water and sonicated. The mixture was spun-down (4000 rpm) for 30 min. The end product was recovered by vacuum filtration and dried under vacuum. The mechanism for PdNPs modified in rGO is displayed in scheme IV in S1 File.

### Suzuki coupling reaction with PdNP/rGO

A solution of aryl halide (0.32 mM) in 8 mL of ethanol:water (1:1), phenylboronic acid (0.38 mM), K_2_CO_3_ (0.96 mM), PdNP/rGO (0.96 M) was prepared. The prepared solution was constantly stirred at 180 °C (15 min). The product formed was extracted using ethyl acetate, recrystallized from ethanol, dried over anhydrous Na_2_SO_4_, and characterized by ^1^H NMR (300 MHz, CDCl_3_), and ^13^C NMR (75.5 MHz, CDCl_3_).

### Characterization methods

PdNP-modified graphene sheets were characterized by UV-Vis absorption spectra, recorded between 200 and 900 nm with a Perkin Elmer Lambda 900 spectrophotometer. FTIR spectra were obtained with a Nicolet 370 FT/IR spectrometer. X-ray diffraction (XRD) analysis was done by a Philips powder diffractometer with PW 1373 goniometer (Cu Ka = 1.5406 Å). Raman spectroscopy was performed using an Alpha 300R, WITEC system. The morphological features of the PdNP/rGO were confirmed by transmission electron microscopy (TEM).The results of the catalytic reactions were recorded using ^1^H and ^13^C NMR collected on a Brüker Avance 200 or 300 MHz spectrometer in CDCl_3_ with TMS as an internal standard; chemical shifts are expressed in parts per million.

## Results and discussion

**Fig 1:**
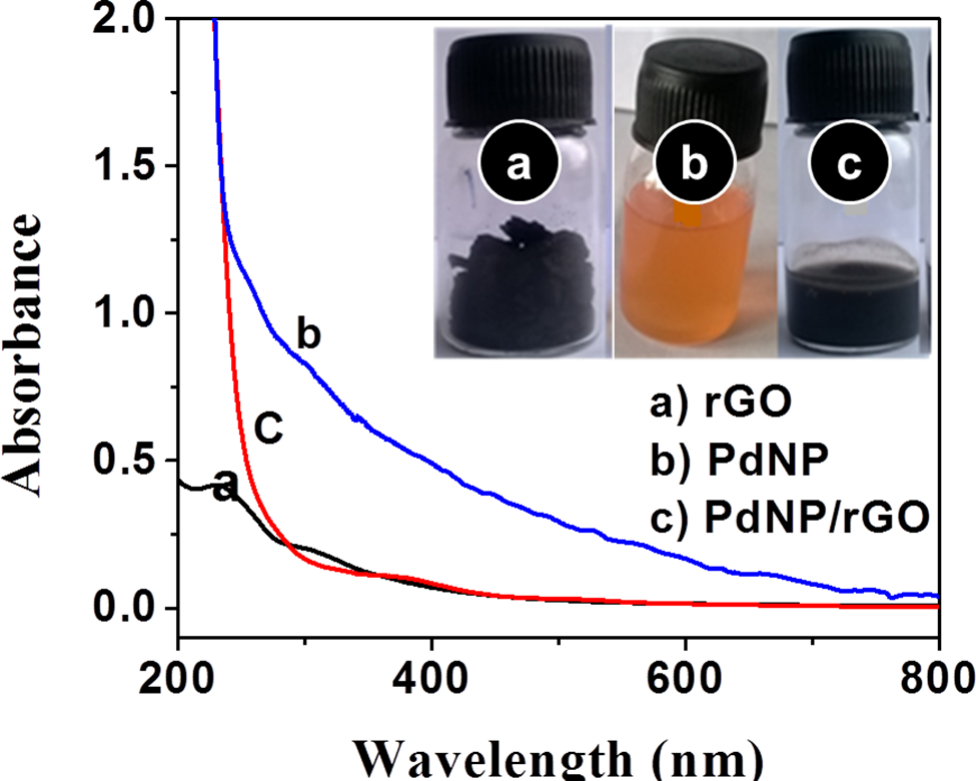
UV-Vis spectroscopic analysis. a) rGO; b) PdNP; andc) PdNP/rGO. Figure inset shows the real samples. Scanned from the wavelength from 200 to 800 nm. The absence of an absorption band at 234 nm shows complete reduction of Pd ions and the formation of PdNP and PdNP/rG.

Palladium nanoparticle (PdNP) and reduced graphene oxide (rGO) are potential nanomaterials, and their synthetic applications are studied [57]. We have synthesized rGO using the method described by Hummers and Offeman using *Ficus carica* fruit extract for the simultaneous chemical reduction of rGO and H_2_PdCl_4_. The progress of the chemical reduction of graphene and subsequent deposition of palladium on the graphene surface was tested by UV-visible spectroscopy. Fig 1 displays the UV-Vis spectra for rGO, *Ficus carica* fruit extract with H_2_PdCl_4_, and PdNPs modified with rGO. The absorption band (a) at 234 nm is attributed to rGO. The absence of an absorption band at 234 nm shows a complete reduction of Pd ions and the formation of PdNP and PdNP/rGO (Fig 1B and 1C).

### Morphological and structural characterization of PdNP/rGO

**Fig 2:**
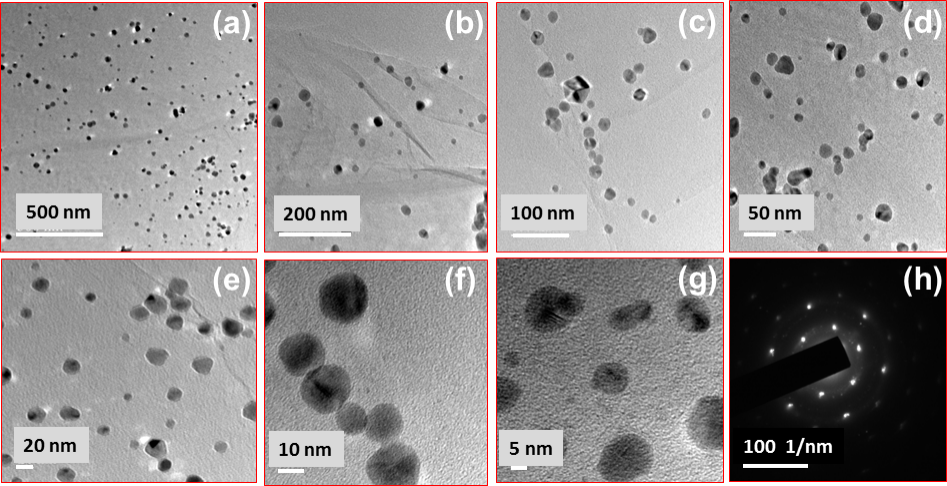
Transmission electron microscopy images. (a-e) Images along with particle size distribution; (g) HRTEM image along with fringe spacing; and (f) SAED image of the PdNP/rGO.

Transmission electron microscopy (TEM) was used to characterize the morphology of PdNP/rGO. The micrographs are shown in Fig 2A–2E) confirm the uniformity of the distribution of PdNP on the rGO nanosheet surfaces, and the immobilized particles are spherical. The crystal lattice of PdNP and the edges of the rGO nanosheets are clearly seen in the images acquired by HRTEM (Fig 2F) and are formed to be ~16 nm. The particle size was measured from the distribution curve and measured average to be ~5 nm. Diffraction dots were captured in the SAED image (Fig 2G), and confirmed the crystallinity of the PdNP.

**Fig 3:**
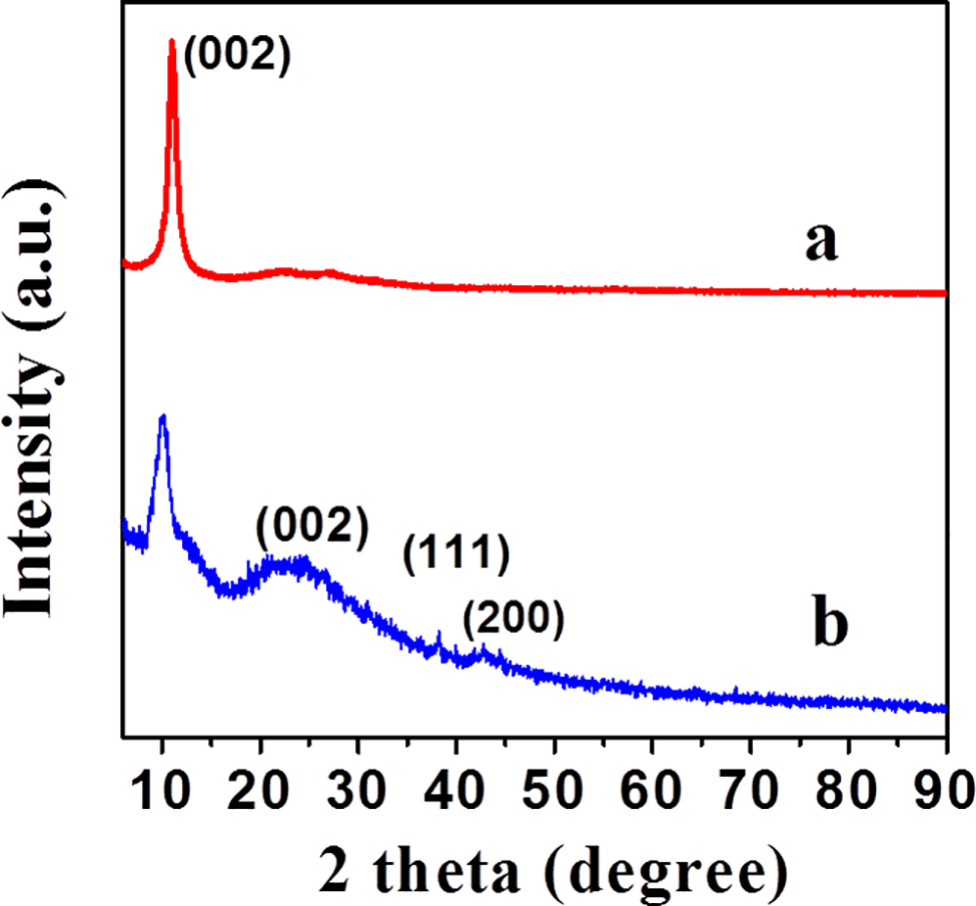
XRD analysis. Patterns of rGO and PdNP/rGO are shown. XRD patterns of PdNP/rGO show a broad peak appeared at 23.2^o^ (002). PdNP/rGO display diffraction peaks at 38.2^o^ and 43.4^o^.

Furthermore, the crystalline structures of PdNP on rGO sheets were confirmed by X-ray diffraction (XRD) analysis, and the obtained data are displayed in Fig 3. Typically, graphite consists of an intense reflection peaks at 26.4^o^ (002), which is moved to the lower Bragg angle in the case of rGO at 10.2° corresponding to (001) plane with d-spacing of 0.81 nm, due to the addition of various oxygen-containing functional groups between the graphite layers during the oxidation process [53]. However, in the XRD patterns of PdNP/rGO, a broad peak appeared at 23.2^o^ (002), whereas the reflection corresponding to (001) plane of rGO at 10.2^o^ disappeared. This observation confirms the destruction of the regular layered structure of rGO and the formation of a few layer rGO sheets in PdNP after the reduction with *Ficus carica*. Moreover, the PdNP/rGO display diffraction peaks at 38.2^o^ and 43.4^o^ corresponding to (111) and (200) planes which can be attributed to the face-centered cubic (*fcc)* structure of PdNP on the surface of rGO (JCPDS card 05–0681, 65–2868, 65–2870, 04–0783).

**Fig 4:**
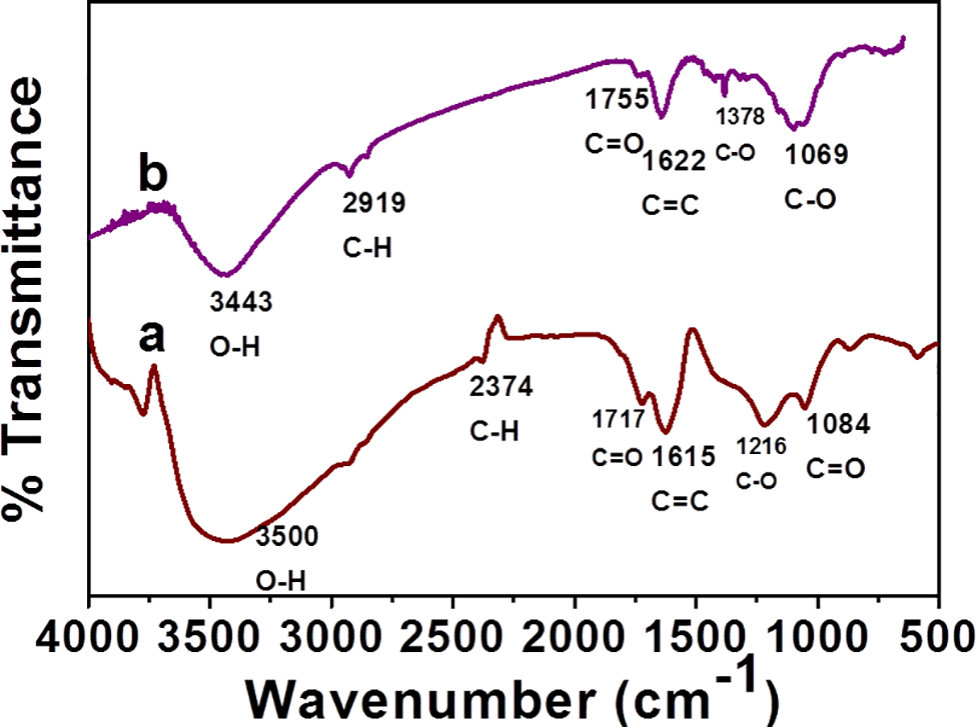
FT-IR analysis. Spectra of a) rGO and b) PdNP/rGO. In the spectrum of rGO, the wide peak observed at 3500 cm^-1^. In the spectra of PdNP/rGO, the peaks at 1622 and 1755 cm^-1^ are corresponding to the disappearance of rGO. The broad band at 3443 cm^-1^ for hydroxyl groups. The peak around 2919 cm^-1^ indicates the formation of C-H stretching vibration. Peaks around 1755 and 1622 are the typical aromatic peaks for the C-H and C-C stretching, respectively.

FTIR spectroscopy was performed to elucidate the occurrence of various chemical groups and/or the phytochemical components in the rGO and PdNP/rGO. Fig 4 shows both FTIR spectra of rGO and PdNP/rGO. In the spectrum of rGO, the wide peak observed at 3500 cm^-1^ corresponds to free OH, where the peaks at 2374, 1717, 1615, 1216 and 1084 cm^-1^, suggest the presence of C-H, C = O, C = C and C-O groups respectively. Typically, the FTIR peaks belonging to the rGO either disappear or their intensities are significantly reduced after the reduction process, which in turn confirms the formation of PdNP modified rGO. In the FTIR spectra of PdNP/rGO, the peaks at 1622 (for C = C stretching) and 1755 cm^-1^ (for C = O stretching) corresponding to the disappearance of rGO, whereas the intensities of some of the peaks are relatively reduced. The broadband at 3443 cm^-1^ for hydroxyl groups shows the reduction of rGO to PdNP/rGO. The peak around 2919 cm^-1^ indicates the formation of C-H stretching vibration. Furthermore, the peak around 1755 and 1622 are the typical aromatic peaks for the C-H and C-C stretching respectively. Where, the peaks corresponding to the C-O stretching of carboxylic acids, alcohols, ether and ester groups appeared at 1378 and 1069 cm^-1^. On the basis of FT IR data, we could infer that phenolic hydroxyl groups of flavones present in the *Ficus carica* fruit extract which played a vital role in the reduction of Pd(II) ions and have strong ability to bind with PdNP. The fruit extract not only acted as a reducing agent but also function as a stabilizing agent by attaching to the surface of rGO sheets.

**Fig 5:**
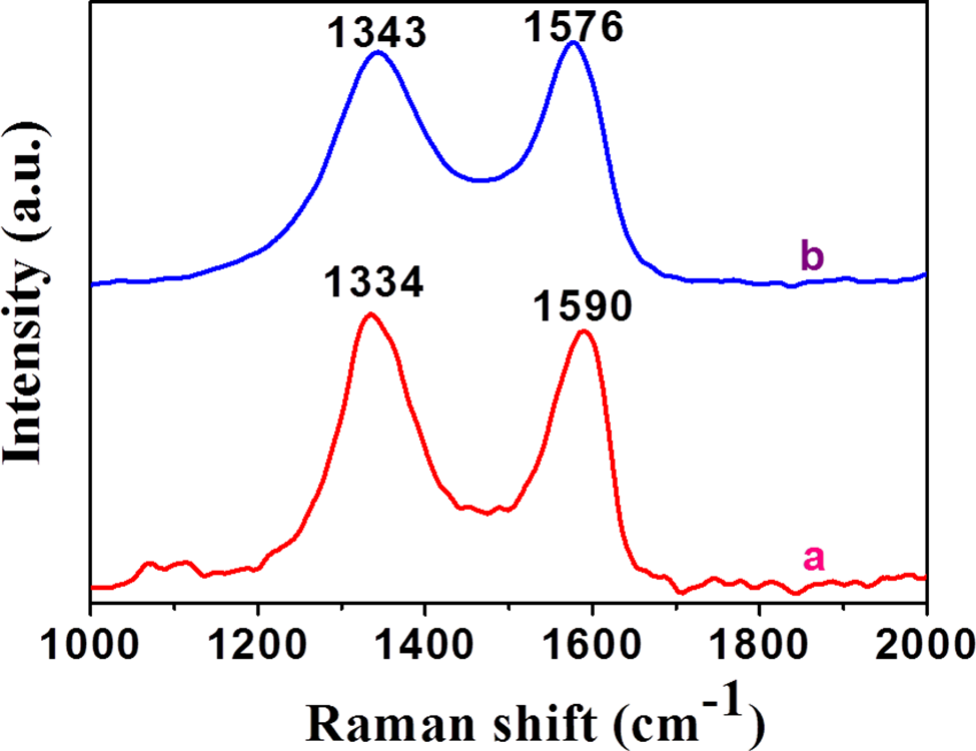
Raman spectroscopic analysis. Spectra of rGO and PdNP/rGO are shown. G and D bands situated at 1576 and 1343 cm^-1^, respectively. Bands are relocated after the reduction with *Ficus carica* in the Raman spectrum of PdNP/rGO. The G band is shifted from 1590 to 1576 cm^-1^ and the D band is relocated from 1334 to 1343 cm^-1^.

Significant changes in the structure of the graphitic substrate during the synthesis were observed by the Raman spectra (Fig 5). Raman spectroscopy is commonly used to determine the amount of order or changes in the structures of carbonaceous compounds. Generally, the graphene consists of two different characteristic signals, with the G and D bands situated at 1576 and 1343 cm^-1^, respectively. Hence, after the oxidation of graphene, the sp^2^ character was destroyed and various defects are formed in rGO, due to which the characteristic bands of graphene, in this case, shifted to 1590 and 1334 cm^-1^. These bands are relocated after the reduction with *Ficus carica* in the Raman spectrum of PdNP/rGO. The G band is shifted from 1590 to 1576 cm^-1^ and the D band is relocated from 1334 to 1343 cm^-1^. These visible changes indicate the reduction rGO with *Ficus carica* and clearly indicate the formation of PdNP on the surface of rGO.

### Catalytic activity of PdNP/rGO for Suzuki cross-coupling reaction

We have carried out Suzuki cross-coupling reaction using PdNP/rGO as a catalyst. The application of catalyst for the synthesis proved very fruitful for a variety of reaction. Initially, we carried out the coupling reaction with different amounts of the catalyst with varying temperatures and solvents to find the optimal conditions. As expected, the product did not form in the absence of a catalyst (Table 1, entry 1). It was noticed that the reaction took place in the presence of a catalyst and the product formed was in good yield in short duration of time. Of the different bases tested (K_2_CO_3_, Na_2_CO_3_ and KOH), the use of PdNP/rGO in water and ethanol (1:1) with K_2_CO_3_ led to the highest conversion (Table 1, entry 5). To further optimize the reaction, various amounts of PdNP/rGO were utilized for the synthesis of biphenyls from two different aryl halides (0.32 mM) and phenylboronic acid (0.38 mM). The best result was obtained by using 0.96 mol% of PdNP/rGO and 0.96 mM K_2_CO_3_ in H_2_O:EtOH at 180°C (Table 1, entry 5). Decreasing the catalyst loading from 0.96 to 0.5 mol% reduced the product yield (Table 1, entry 8). There was no increase in the product yield when more catalyst was used and 0.09 mol % was found to be the optimum concentration to set maximum yield of the product.

**Table 1:** Optimization of conditions for the Suzuki–Miyaura coupling of aryl halides with phenylboronic acid.

Entry	PdNP/rGO(mol %)	Base	Temperature (°C)	Yield (%)
1	0	KOH	100	0
2	1	KOH	100	85
3	1	Na_2_CO_3_	100	87
3	1	K_2_CO_3_	100	92
4	0.96	K_2_CO_3_	100	94
5	0.96	K_2_CO_3_	180	98
6	0.96	K_2_CO_3_	80	83
7	0.96	K_2_CO_3_	45	78
8	0.5	K_2_CO_3_	180	95
9	0.96	K_2_CO_3_	200	98

We examined the use of PdNP/rGO with aryl halides containing electron-donating and electron-withdrawing groups. Our method is general and can tolerate various kinds of aryl halides. This strategy afforded the expected products in good yields. The general reaction scheme is shown in (Scheme I in S1 File). The progression of the catalytic reaction was followed by NMR studies, which confirmed that the reaction was complete within 15 min (MS, ^1^H NMR and ^13^C NMR spectra are shown in S1 File. The required time for the reaction to attain the stage of completion (15 min) is much shorter than those of other reported methods [58–62], and the catalyst in this system is easier to recover and can be used repeatedly without any significant loss in activity. The general mechanism for the PdNP/rGO catalyzed C-C coupling is shown in Scheme II in S1 File.

### Catalyst recyclability

**Fig 6:**
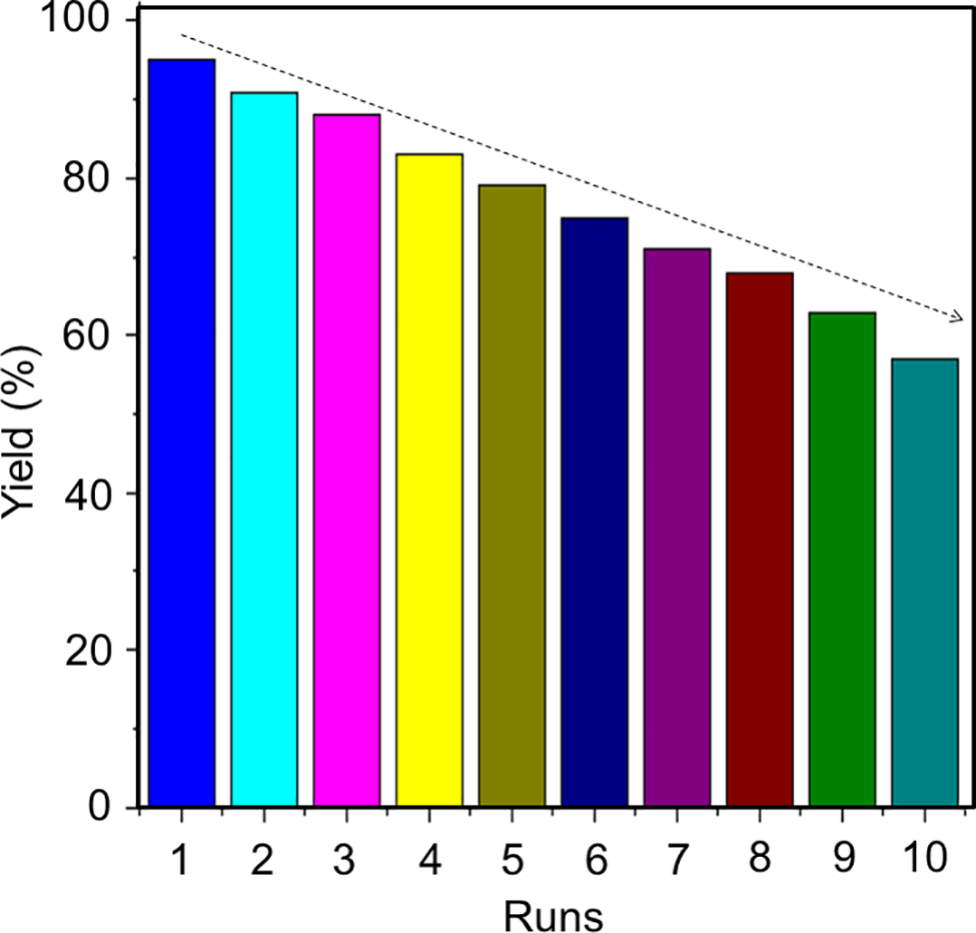
Recycling of PdNP/rGO. Data were obtained after repeated uses. Ten runs were performed and it displays the consecutive usages of 95%, 91%, 88%, 83%, 79%, 75%, 71%, 68%, 63% and 57%.

Recovery and reuse of the catalyst are significant issues in Suzuki coupling reactions. Furthermore, facile separation and recycling make the catalyst make it more useful in industrial applications. When the reaction completed, PdNP/rGO was partitioned from the reaction solution by spinning-down. The residue was thoroughly washed using water followed by ethanol, dried, and reused. The activities of ten consecutive usages, 95%, 91%, 88%, 83%, 79%, 75%, 71%, 68%, 63% and 57%, showed the recyclability of the catalyst (Fig 6). This recyclability indicates the catalyst is very stable and has a high turnover rate under the reaction conditions.

Moreover, we compared the performance of the current Pd/rGO catalysts with other Pd nanoparticle catalysts developed for cross-coupling reactions. There are many examples in the literature of Pd nanoparticles stabilized by capped polymeric materials for cross-coupling reactions, and in particular Suzuki reactions [63]. Although those nanocatalysts demonstrated reactivity toward Suzuki cross-coupling reactions, they exhibit limited catalytic activity such as lower product yields, lack of efficient recyclability and low TON and TOF.

**Fig 7:**
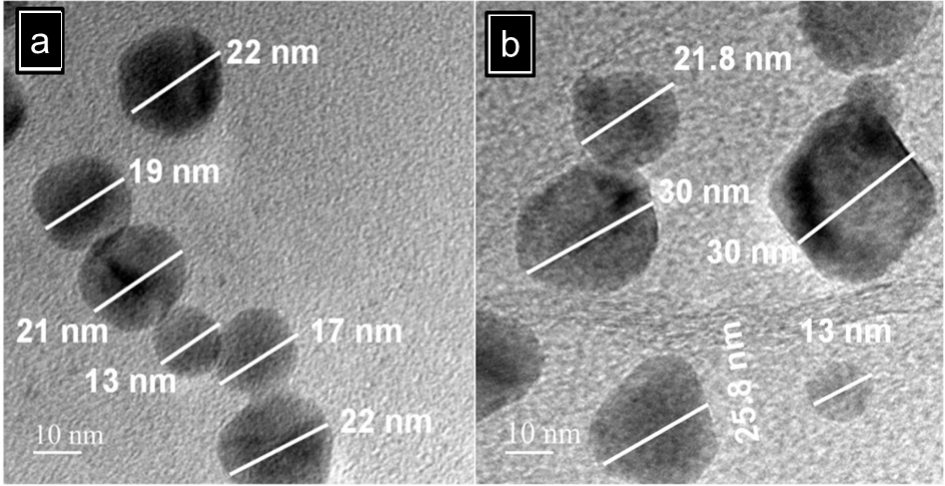
TEM images for recycling of PdNP/rGO. a) before and b) After five consecutive cycles. The degree of agglomeration of catalyst does not show the significant change between the first and the fifth runs.

Fig 7 compares the TEM images of PdNP/rGO, before and after the use of 5 consecutive cycles. It is clear that the degree of agglomeration of the catalyst does not change much between the first and the fifth runs consistent with the ability to efficiently recycle this catalyst without loss of activity. It can be concluded that the current Pd/PRGO catalyst is highly stable and can be used as efficient catalysts for cross-coupling reactions as compared to other Pd nanoparticles stabilized by polymeric molecules.

## Conclusions

We have developed palladium nanoparticle (PdNP)-modified reduced graphene oxide (rGO) sheets by a one-pot method using *Ficus carica* fruit juice as the reducing agent. The PdNP/rGO catalyst exhibits enhanced catalytic activity and good reusability in Suzuki cross-coupling reactions. This research opens up a novel method for the *in situ* generation of PdNP on rGO nanosheets and is an eco-friendly method for the cross coupling reaction. This greener approach may find applications in various medicinal as well as technological fields, and the catalyst produced by this method can be reused without significant loss of activity [64–67]. We evidenced the wide usage of this methodology, and it will help in the generation of advanced catalytic materials.

## Supporting information

S1 File **Scheme I:** PdNPs/RGO catalyzed Suzuki-Miyura cross-coupling of aryl halides with various phenylboronic acids. **Scheme II:** Mechanism for the PdNPs/RGO catalyzed C–C coupling reactions. **Scheme III:** The mechanism for reduction of Pd ions using *Ficus carica* fruit extract. **Scheme IV:** Mechanism for PdNPs modified in reduced graphene oxide. MS, ^1^H NMR and ^13^C NMR spectroscopic data are reported.
(DOCX)
