# Supplementary material for: Palladium nanoparticle-decorated reduced graphene oxide sheets synthesized using Ficus carica fruit extract: A catalyst for Suzuki cross-coupling reactions
Source: PLoS One. 2018 Feb 21;13(2):e0193281. doi: 10.1371/journal.pone.0193281 (PMC5821401; doi:10.1371/journal.pone.0193281)
Supplement: S1 File — Scheme I: PdNPs/RGO catalyzed Suzuki-Miyura cross-coupling of aryl halides with various phenylboronic acids. Scheme II: Mechanism for the PdNPs/RGO catalyzed C–C coupling reactions. Scheme III: The mechanism for reduction of Pd ions using Ficus carica fruit extract. Scheme IV: Mechanism for PdNPs modified in reduced graphene oxide. MS, 1H NMR and 13C NMR spectroscopic data are reported. (DOCX) [file pone.0193281.s001.docx]

**Supplementary Information**

**Palladium Nanoparticle-Decorated Reduced Graphene Oxide Sheets Synthesized Using *Ficus Carica* Fruit Extract:**

**A Catalyst for Suzuki Cross-coupling Reactions**

Jaculin Raiza Anasdass^1^, Pandian Kannaiyan^1*^,

Raghunathan Raghavachary^2^, Subash C.B. Gopinath^3,4^, Yeng Chen^5,6^

^1^Department of Inorganic Chemistry, University of Madras, Chennai, India.

^2^Department of Organic Chemistry, University of Madras, Chennai, India.

^3^School of Bioprocess Engineering, Arau, Universiti Malaysia Perlis, Perlis, Malaysia.

^4^Institute of Nano Electronic Engineering, Kangar, Universiti Malaysia Perlis, Perlis, Malaysia.

^5^Department of Oral & Craniofacial Sciences, Faculty of Dentistry, University of Malaya, Kuala Lumpur, Malaysia.

^6^Oral Cancer Research & Coordinating Center (OCRCC), Faculty of Dentistry, University of Malaya, Kuala Lumpur, Malaysia.

**
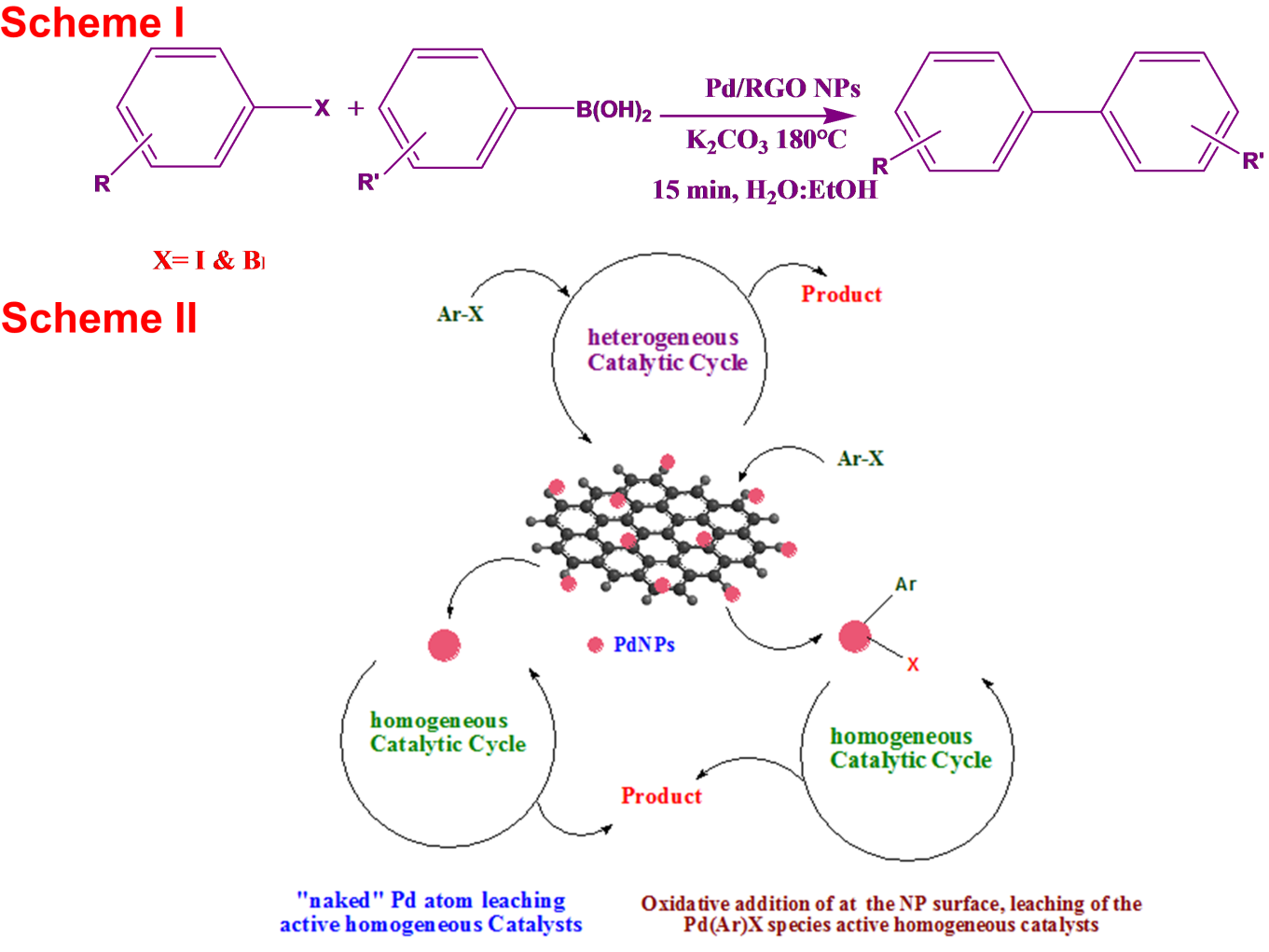
**

**Scheme III**

**
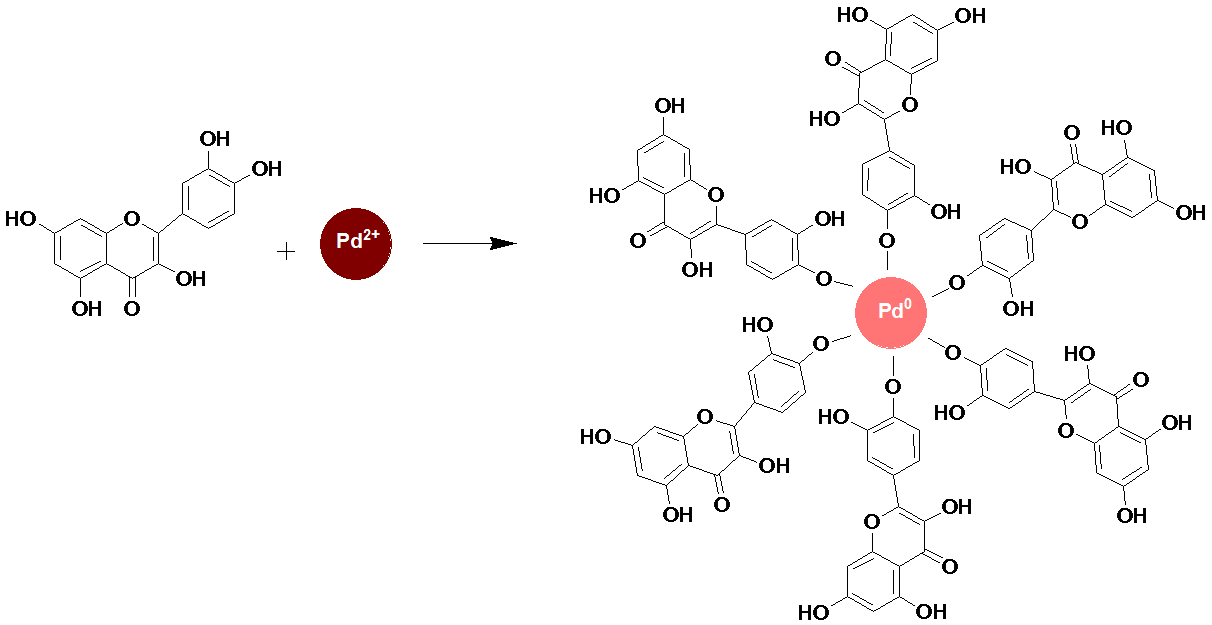
**

**Scheme IV**

**
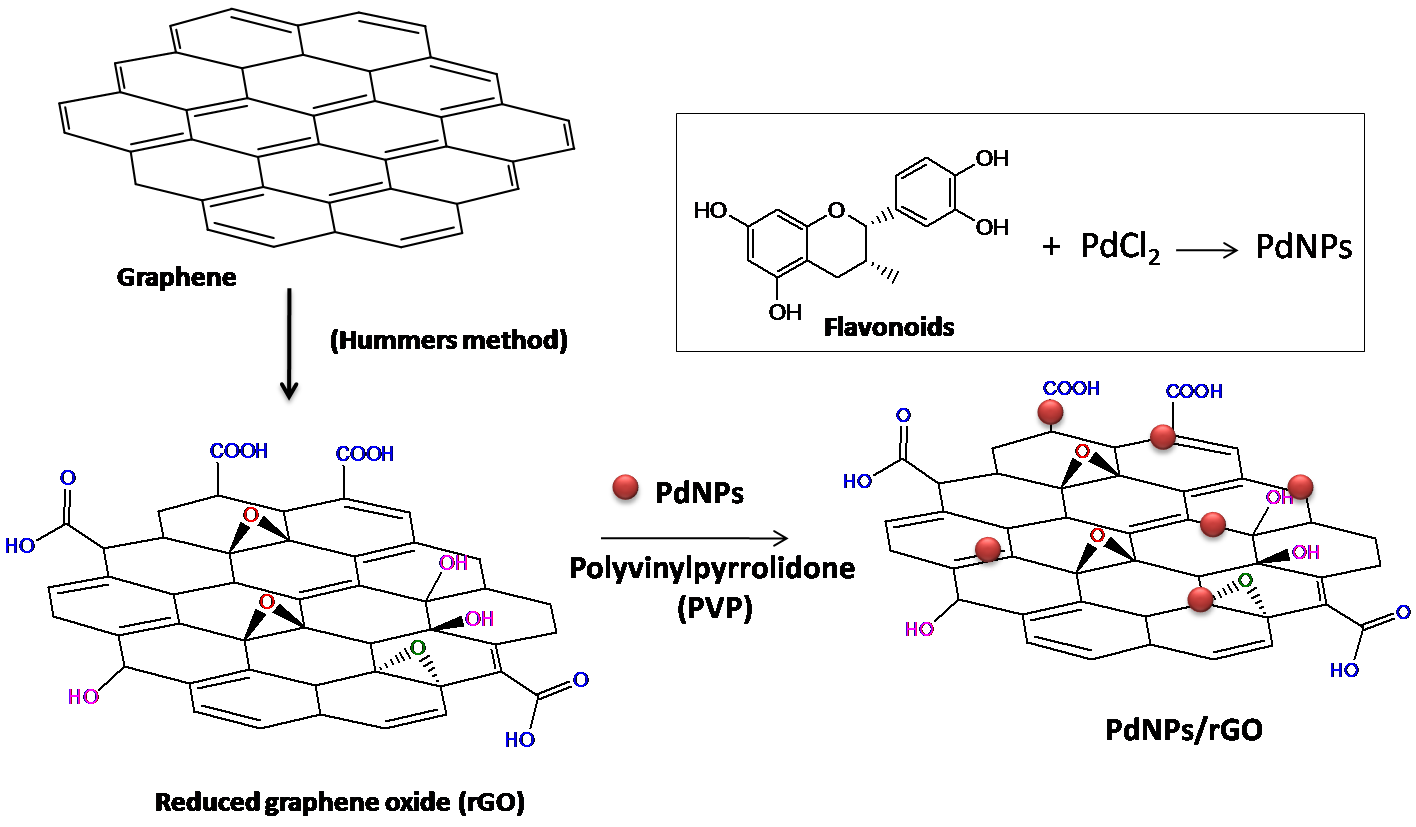
**

**NMR Spectral data**

**4'-Nitrobiphenyl-2-carbonitrile (1)**

The 4'-nitrobiphenyl-2-carbonitrile synthesized as pale yellow solid in 97% yield from 1-iodo-4 nitrobenzene (50mg, 0.32mM, 1 equiv) and 2-cyanophenyl boronic acid (47mg, 0.38mM, 1.2 equiv). ^1^H NMR (300MHz CDCl_3_): *δ*_H_ 7.28-7.94 (m, 8H). ^13^C NMR (75 MHz, CDCl_3_): *δ*_C_ 103.6, 122.4, 128.1, 128.7, 130.1, 132.2, 133.9, 143.6, 147.7. MS(EI): m/z 225.6 M^+^.

**3-Chlorobiphenyl (2)**

The 3-chlorobiphenyl synthesized as pale yellow solid in 90% yield from 1-bromobenzene (50mg, 0.32mM, 1 equiv) and 3-chlorophenyl boronic acid (47mg, 0.38mM, 1.2 equiv). 1H NMR (300MHz CDCl_3_): δ_H_ 7.31 – 7.38 (m, 3H), 7.47 (d, 3H J = 7.5Hz); 7.97 (d, 2H J = 7.2Hz); 8.01 (s, 1H). ^13^C NMR (75 MHz, CDCl_3_): *δ*_C_ 126.7, 128.1, 129.6, 132.9, 133.6, 134.4, 135.3. MS (EI): m/z 188.7 M^+^.

**2,6-Dimethoxy-4'-nitrobiphenyl (3)**

The 2,6-dimethoxy-4'-nitrobiphenyl synthesized as pale yellow solid in 95% yield from 1-iodo-4 nitrobenzene (50mg, 0.32mM, 1 equiv) and 2,6 dimethoxy phenyl boronic acid (47mg, 0.38mM, 1.2 equiv). 1H NMR (300MHz CDCl_3_): δ_H_ 3.92 (s, 6H, OCH_3_); 6.64 (d, 1H J = 8.4 Hz); 7.89-7.96 (m, 6H). ^13^C NMR (75 MHz, CDCl_3_): *δ*_C_ 56.1, 102.7, 104.4, 124.8, 133.0, 138.7, 143.4, 147.7, 165.5. MS (EI): m/z 260.1 M^+^.

**2',6'-Dimethoxybiphenyl-4-carbonitrile (4)**

The 2',6'-dimethoxybiphenyl-4-carbonitrile synthesized as pale yellow solid in 92% yield from 4-iodobenzonitrile (50mg, 0.32mM, 1 equiv) and 2,6 dimethoxy phenyl boronic acid (47mg, 0.38mM, 1.2 equiv). ^1^H NMR (300MHz CDCl_3_): δ_H_ 3.96 (s, 6H, OCH_3_); 6.64(d, 2H J = 8.4 Hz); 7.36-7.43 (m, 3H); 7.85 (d, 2H J = 7.8 Hz).^13^C NMR (75 MHz, CDCl_3_): δc 56.1, 100.3, 104.4, 111.7, 118.2, 133.0, 133.2, 138.5, 165.5. MS (EI): m/z 240.7 M^+^.

**2,6-Dimethoxybiphenyl (5)**

The 2,6-dimethoxybiphenyl synthesized as pale yellow solid in 94% yield from 1-bromobenzene (50mg, 0.32mM, 1 equiv) and 2,6 dimethoxy phenyl boronic acid (47mg, 0.38mM, 1.2 equiv). ^1^H NMR (300MHz CDCl_3_): δ_H_ 3.91 (s, 6H); 6.63 (t, 3H J = 8.4 Hz); 7.24(t, 3H J=5.4 Hz); 7.40 (t, 2H J=8.4 Hz).^13^C NMR (75 MHz, CDCl_3_): δc 56.0, 106.4, 110.5, 127.8, 130.2, 135.3, 160.4. MS (EI): m/z 215.6 M^+^.

**3-Chlorobiphenyl (6)**

The 3-chlorobiphenyl synthesized as colorless solid in 92% yield from iodobenzene (50mg, 0.32mM, 1 equiv) and 3-clorophenyl boronic acid (47mg, 0.38mM, 1.2 equiv). ^1^H NMR (300MHz CDCl_3_): δ_H_ 7.43 (t, 3H J = 7.5 Hz); 7.55 (d, 2H J = 7.8 Hz); 8.03 (s, 2H); 8.07 (d, 2H J = 9 Hz). ^13^C NMR (75 MHz, CDCl_3_): δc 56.0 (OCH_3_); δc 126.3, 127.8, 129.6, 133.0,133.6, 134.4, 135.2. MS (EI): m/z 188.9 M^+^.

**2, 4’, 6-Trimethoxybiphenyl (7)**

The 2,4',6-trimethoxybiphenyl synthesized as pale yellow solid in 93% yield from 4-iodoanisole (50mg, 0.32mM, 1 equiv) and 2,6 dimethoxy phenyl boronic acid (47mg, 0.38mM, 1.2 equiv). ^1^H NMR (300MHz CDCl_3_): δ_H_ 3.91 (s, 9H); 6.65 (t, 4H J = 12 Hz); 7.37 (s, 2H); 7.38-7.56 (m, 1H). ^13^C NMR (75 MHz, CDCl_3_): δc 56.2, 60.8, 102.7, 110.6, 112.4, 124.8, 138.7, 147.7, 152.9. MS (EI): m/z 245.2 M^+^.

**3, 4, 5-Trimethoxy-4'-nitrobiphenyl (8)**

The 3,4,5-trimethoxy-4'-nitrobiphenyl synthesized as pale yellow solid in 93% yield from 1-iodo-4 nitrobenzene (50mg, 0.32mM, 1 equiv) and 3,4,5 trimethoxy phenyl boronic acid (47mg, 0.38mM, 1.2 equiv). ^1^H NMR (300MHz CDCl_3_): δ_H_ 3.89 (s, 3H); 3.98 (d, 6H J = 9.6 Hz); 7.89-7.96 (m, 6H). ^13^C NMR (75 MHz, CDCl_3_): δc 56.1, 56.4, 104.3, 120.8, 127.5, 131.4, 137.9, 143.2, 147.9, 152.8. MS (EI): m/z 290.4 M^+^.

**2,4-Dichloro-4'-methoxybiphenyl (9)**

The 2,4-dichloro-4'-methoxybiphenyl synthesized as colorless solid in 93% yield from 4-iodoanisole (50mg, 0.32mM, 1 equiv) and 2,4 dichloro phenyl boronic acid (47mg, 0.38mM, 1.2 equiv). ^1^H NMR (300MHz CDCl_3_): δ_H_ 3.48 (s, 3H); 7.26-7.39 (m, 5H); 7.89 (d, 2H J = 8.1 Hz) ^13^C NMR (75 MHz, CDCl_3_): δc 56.1, 115.2, 126.4, 129.2, 131.3, 134.9, 135.8, 160.3. MS (EI): m/z 255.3 M^+^.

**
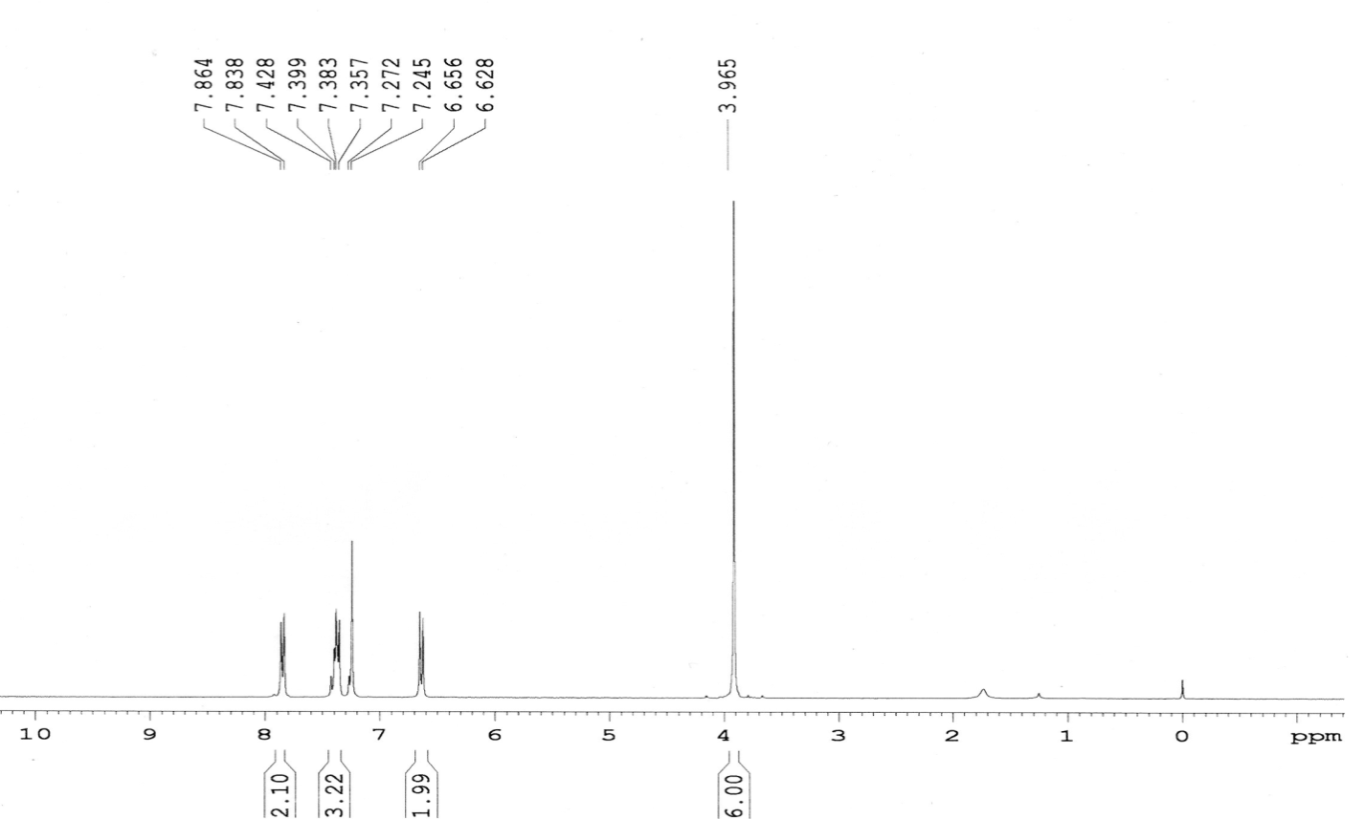
^
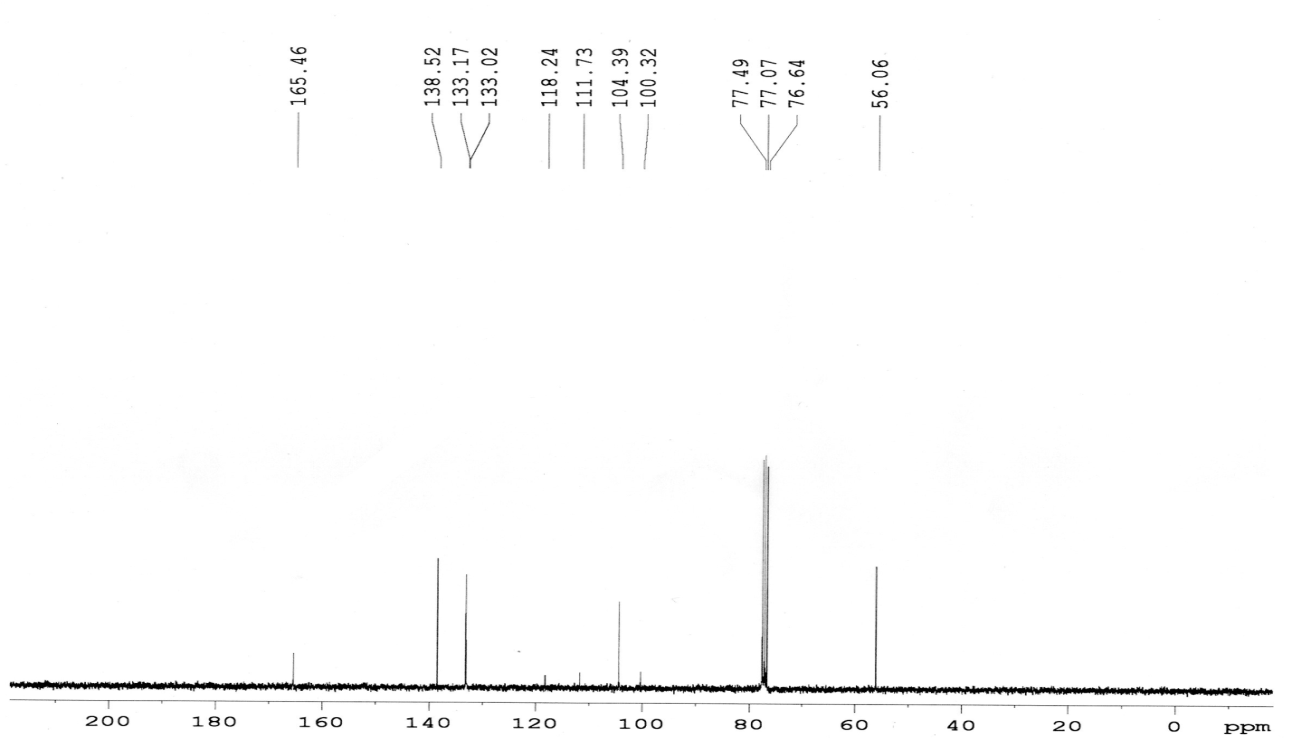
1^H NMR spectrum (300MHz, CDCl_3_) of compound 4**

 **(4)**

**^13^C NMR spectrum (75MHz, CDCl_3_) of compound 4**

**
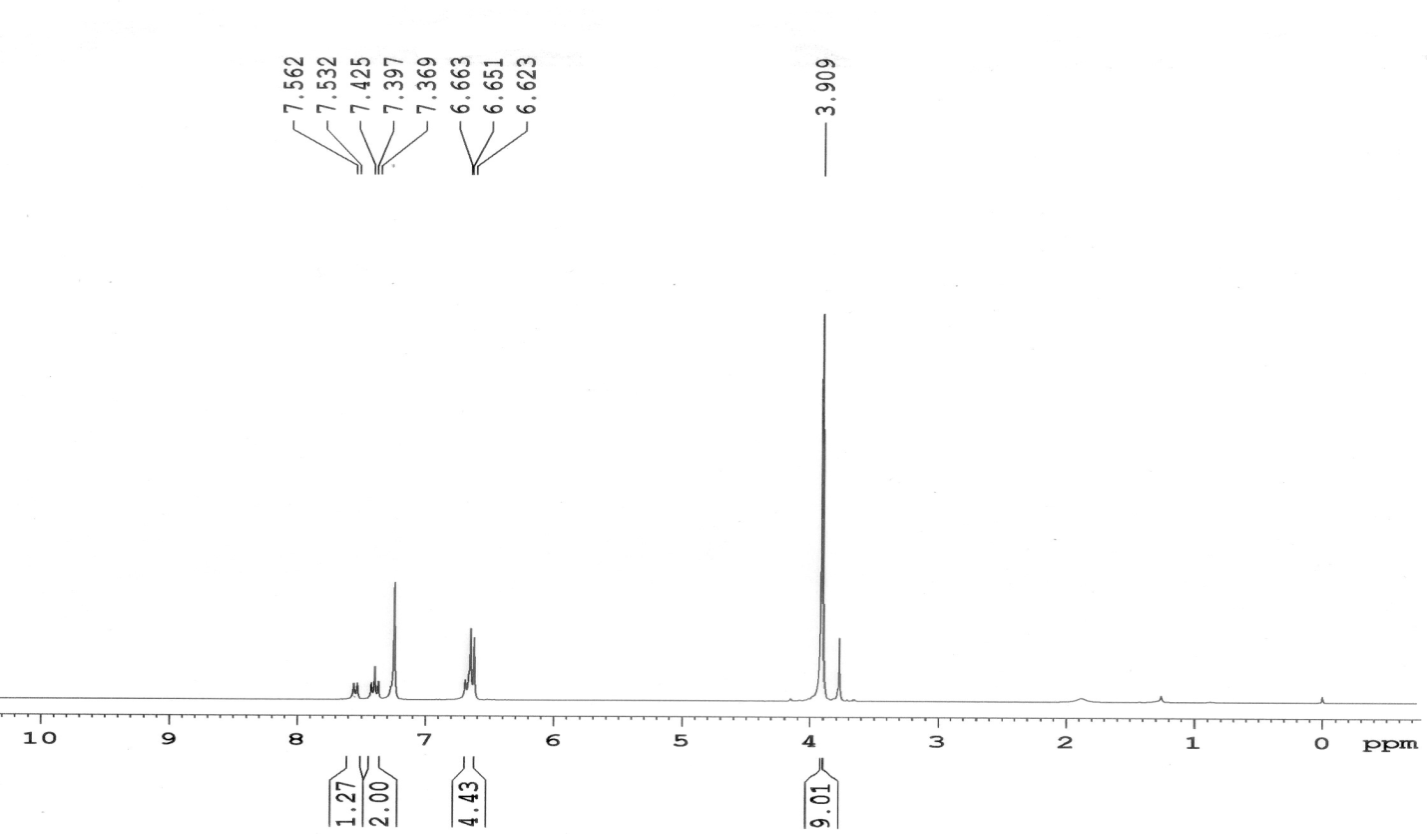
**

 **(7)**

**^
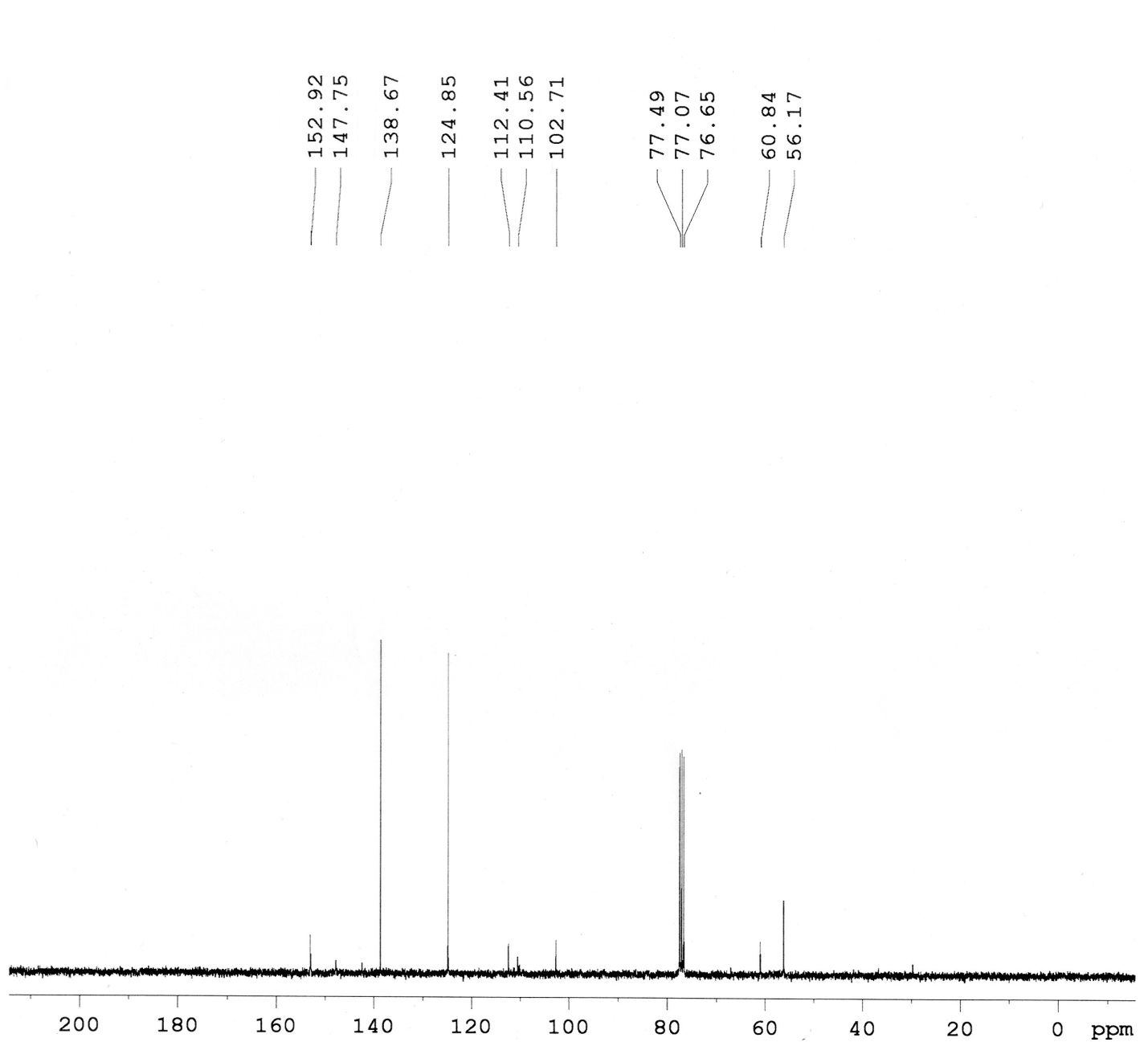
1^H NMR spectrum (300MHz, CDCl_3_) of compound 7**

**^13^C NMR spectrum (75MHz, CDCl_3_) of compound 7**

**Selected Mass Spectra**


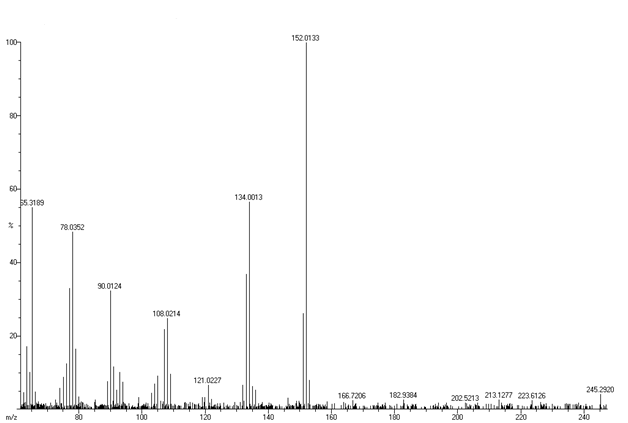

 **(7)**

**
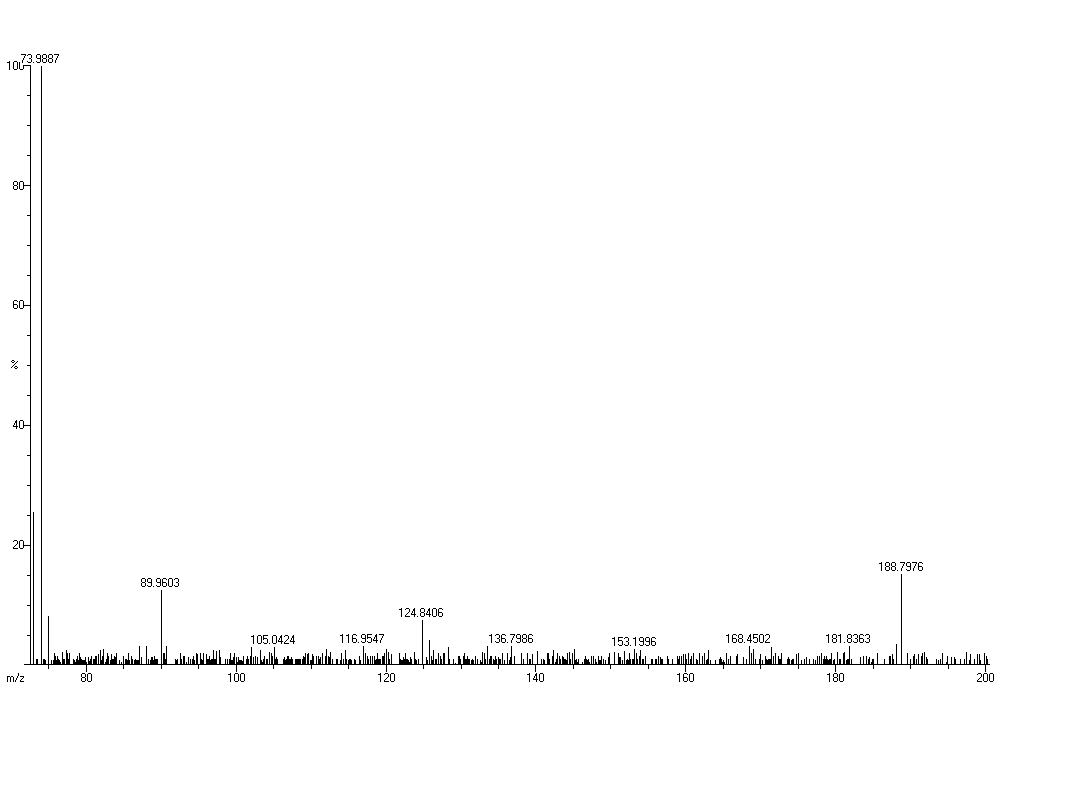
EI mass spectrum of compound 7**

 **(2)**

**EI mass spectrum of compound 2**
